# Genome-wide association study on abdomen depth, head width, hip width, and withers height in native cattle of Guilan (*Bos indicus*)

**DOI:** 10.1371/journal.pone.0289612

**Published:** 2023-08-18

**Authors:** Mohammad Golshani Jourshari, Abdol Ahad Shadparvar, Navid Ghavi Hossein-Zadeh, Farjad Rafeie, Mohammad Hossein Banabazi, Anna Maria Johansson

**Affiliations:** 1 Department of Animal Science, Faculty of Agricultural Sciences, University of Guilan, Rasht, Iran; 2 Department of Agricultural Biotechnology, Faculty of Agricultural Sciences, University of Guilan, Rasht, Iran; 3 Department of Biotechnology, Animal Science Research Institute of IRAN (ASRI), Agricultural Research, Education & Extension Organization (AREEO), Karaj, Iran; 4 Department of Animal Breeding and Genetics (HGEN), Centre for Veterinary Medicine and Animal Science (VHC), Swedish University of Agricultural Sciences (SLU), Uppsala, Sweden; Sun Yat-Sen University, CHINA

## Abstract

Native breeds in any country are a national capital, and their preservation is of great importance. Native Cattle of Guilan (NCG) is one of the few pure native breeds in Iran and the West Asia region. During the last decade, NCG population has decreased by more than 40%. This study aimed to identify significant single nucleotide polymorphisms (SNPs) and candidate genes associated with meat production traits in NCG using a genome-wide association study (GWAS). The blood and hair samples were collected from 72 NCG individuals and genotyped using the Illumina Bovine SNP50 chip. The results of the genomic scan showed that several SNPs were associated with abdominal depth, head width, hip width, and withers height in NCG. Several candidate genes were identified, including multiple epidermal growth factor-like domains 11 (*MEGF11*), Methionine Sulfoxide Reductase A (*MSRA*), chondroitin sulfate synthase 3 (*CHSY3*), Cyclin-Dependent Kinase 7 (*CDK7*), and Parkin (*PRKN*) genes, which are involved in muscle growth, meat tenderness, differentiation of fat cells, fat metabolism, and adipogenesis. These genes can contribute to meat quantity and quality in NCG. This study provided valuable insights into the genetics of NCG and the identification of effective genes associated with meat production traits. The results of this study could be used for the preservation and sustainable use of this breed of native cattle, as an important genetic resource in Iran.

## Introduction

Native breeds of any country are a national capital, and their preservation is of great value and importance. After thousands of years of natural selection, native livestock have continued to live and reproduce after overcoming adversity and adverse environmental conditions [1].

The vast land of Iran has diverse climates due to special geographical conditions. In these conditions, natural and artificial selection has led to the emergence of breeds of domestic animals with diverse talents in this country. Native animals, after many years of natural selection, have adapted to the environmental conditions and stresses caused by the food limitations of their habitat. Resistance to native and regional diseases is also considered one of the important advantages of these animals. In most cases, natural selection has been made against economic traits in animals that are raised in unfavorable environmental conditions, and it causes these animals not to produce as much as the breeds that were selected in favorable environmental conditions [1].

**Fig 1:**
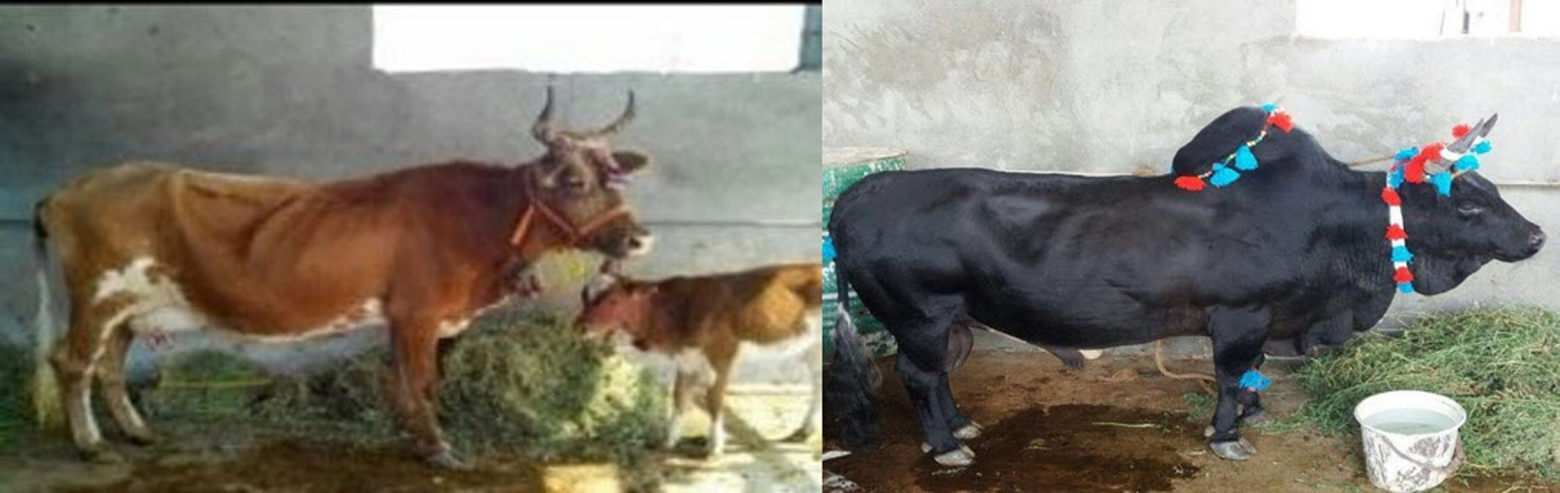
Male (right) and female (left) NCG.

**Fig 2:**
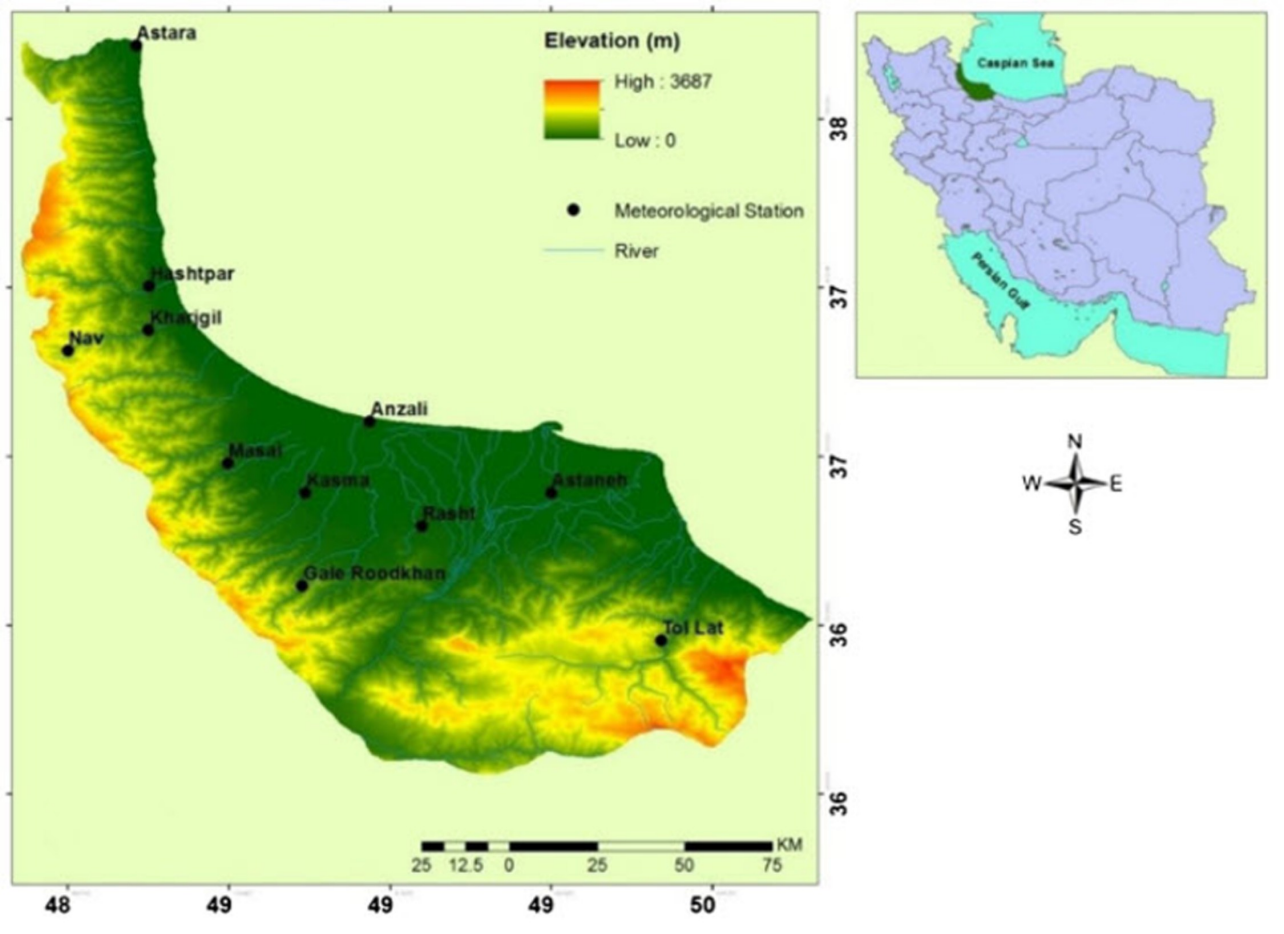
Map of Guilan province illustrating the habitats of NCG.

Native Cattle of Guilan (NCG) is one of the few pure native breeds in Iran and the West Asia region, and according to statistics, during the last decade, its population has decreased by more than 40%. It is believed that these cattle were brought to Iran around 9000 years BC. The NCG has a medium size and withers and a long dewlap (Fig 1), which zoologically belongs to the group of cattle of the Indian subcontinent (*Bos indicus*). NCG currently can be seen in various colors from black to yellow and henna in rural regions of the Guilan province of Iran (Fig 2). The Guilan province is located in the north of the country and lies along the Caspian Sea. This breed is dual-purpose and is much better in meat quality and carcass drop compared to foreign breeds such as Holstein and Simmental and is more welcomed. A noteworthy feature of this breed is the presence of withers, which are found in most native males and are less common in females. The meat of withers has 40 to 60 percent fat and is very tasty, as it is often sold at almost double the price [1].

One of the main objectives of livestock breeders and researchers is to improve growth and carcass traits [1, 2] because these traits account for up to 60–70% of carcass price [1, 3]. Body size is a tool for monitoring animal growth during the fattening period [1, 4]. By controlling the development of each animal, higher profits can be achieved by increasing the effectiveness of diets and management [5, 6].

Sequencing with high throughput has caused single nucleotide polymorphisms (SNPs) to be proposed as technologically developed high-frequency markers in genetics. In addition, the possibility of genotyping hundreds or thousands of animals for this large number of SNPs is provided [7].

SNP genotyping technologies are powerful tools for breeding programs. Genomic selection using SNPs is a new tool for selecting the best animals. In addition, high-density maps using SNPs can provide helpful tools for studying the genetic variance of quantitative traits. It is possible to scan for genetic sequences associated with a specific phenotype. This is called a genome-wide association study (GWAS) and is specifically designed to identify genetic factors related to diseases in humans [8] and economic traits in animals [9]. During the last decade, GWAS has become an important source for generating novel hypotheses in the field of genetics. Therefore, GWASs tend to be suitable for detecting common variants associated with specific phenotypes [10].

One of the most important goals of studying quantitative trait loci is identifying genetic polymorphisms related to the trait. SNPs are single-base pair differences that have overgrown rapidly as molecular markers and have been studied in many species. SNPs have more advantages than other markers, which can be pointed out as accessibility in very high numbers, presence in coding and non-coding regions at the genome level, fewer errors, and ease of comparing results between different studies [11]. One way to detect SNPs is to use commercialized chips for the model species under study (species with whole genomes sequenced). These chips are specific microarrays produced for genotyping the loci of known SNPs.

When a large set of genome-wide scattered polymorphic markers are genotyped, it is possible to probe for genetic sequences associated with a specific phenotype [12]. SNP chips have been designed for several domestic animal species [13], and cattle are one of the best options for using new technologies for genomic selection. Cattle chips include low-density chips (3K and 7K) [14], medium-density chips (50 K) [15], and high-density chips (628 K and 777 K). SNP chips with densities of 50 K and 60 K have also been developed for sheep, pigs [16], and chickens [17].

To conduct a powerful GWAS, a large set of polymorphic markers capable of capturing the desired genetic variation at the genome level is required [18]. This condition is made possible by the use of high-density SNP arrays that are currently implemented for genotyping individuals in GWAS studies. Information about phenotypes must also be available to find associations with genotypes. Phenotypes can be categorical or quantitative. However, if the phenotypic information is quantitative, for example, some measurements, the GWAS will be a quantitative study. The quantitative design seems more powerful, but case-control designs have also been associated with very successful results [8]. In GWAS studies, sample size also plays an important role. Sequences involved in the variation of complex phenotypic traits have small effect sizes, and therefore, large sample sizes are needed to obtain accurate results [18].

Statistical tests in GWAS need to be corrected for factors that can affect the obtained results. These factors include age, gender, and place of study, which can be considered fixed effects in the research. Another essential factor in GWAS studies is the genetic structure of the population under study. In linkage studies, it is assumed that allelic differences are only associated with the desired trait. However, if individuals in a linkage study are from different subpopulations, this allelic difference may be related to the genetic background of the individuals [19]. This issue is called population stratification, and if it is not considered in the surveys, it can cause false positive connections. The main reason for the population stratification of non-random interbreeding among groups is physical separation, which results in the genetic drift of allelic frequency in each group. Therefore, the study of the population structure before applying further studies is necessary, and the correction of the study based on population stratification will be required [20].

In a previous study [21], the genetic polymorphism of the bovine *KLF6* gene and its association with body and carcass traits were evaluated in Qinchuan cattle. The haplotypes and their association with body measurement and carcass quality traits of body length, hip height, hip width, chest depth, chest circumference, ultrasound loin area, and intramuscular fat percentage were identified. These findings provided new insights about KLF6 gene contribution in the marker-assisted selection for growth and carcass characteristics of Qinchuan cattle [21]. To the authors’ knowledge, this is the first GWAS on some major morphometric traits in NCG. Therefore, this study aimed to identify effective genes on abdomen depth, head width, hip width, and withers height in NCG. These genes can be used to identify animals with potential for meat production.

## Materials and methods

A total of 117 heads of mature NCG (between three to four years of age) belonging to eight herds (in Guilan province, Iran) covered by registration and record-keeping, which had production and ancestor records, were sampled. Plastic hollow-pointed syringes with a capacity of 6 cc were used to collect blood from the animals, which were treated with EDTA anticoagulant solution. One syringe was used for each animal. In addition to blood sampling, hair samples were collected from all animals during biometric operation and placed in zip-lock bags, with the animal number recorded on each sample. Moreover, photographs were taken of all animals during biometrics. A three-digit number was assigned to each blood and hair sample. Samples were then transferred to a -20°C freezer for storage during transportation and handling. Animal care and use in this experiment were according to protocols approved by the University of Guilan Institutional Animal Care and Use Committee (#53124 /p 15). To have animals with the same age conditions and minimum familial relationships, 72 out of a total of 117 cattle were sampled. Genomic DNA from 72 samples was extracted from more than 72 hair roots collected using DNeasy Blood and Tissue kit (Qiagen, Germany). Then, samples were genotyped at Neogen GeneSeek Inc. (Lincoln, NE, USA) using the Illumina 50K Bovine SNP BeadChip array (Neogen GeneSeek, Lincoln, NE). Traits evaluated were: abdominal depth, head width, hip width, and withers height. These traits were measured once on each animal by the same evaluator. The measurements were taken using tape and or a measuring stick. Summary statistics for these traits are indicated in Table 1.

**Table 1:** Summary statistics of morphometric traits in the studied population of NCG.

Trait	Mean	SD	CV (%)
Abdomen depth (cm)	56.08	4.47	7.97
Withers height (cm)	14.64	5.39	36.82
Head width (cm)	17.17	2.43	14.15
Hip width (cm)	34.38	3.85	11.20

The following model was considered for association analysis:

y=Xb+Zu+e

where **y** is the vector of phenotypic records, **b** denotes the fixed effects of SNPs with association matrix **X**, **u** is the vector of the random polygenic effect of the individual with the incidence matrix **Z,** and **e** denotes the vector of random residual effects [22].

**Fig 3:**
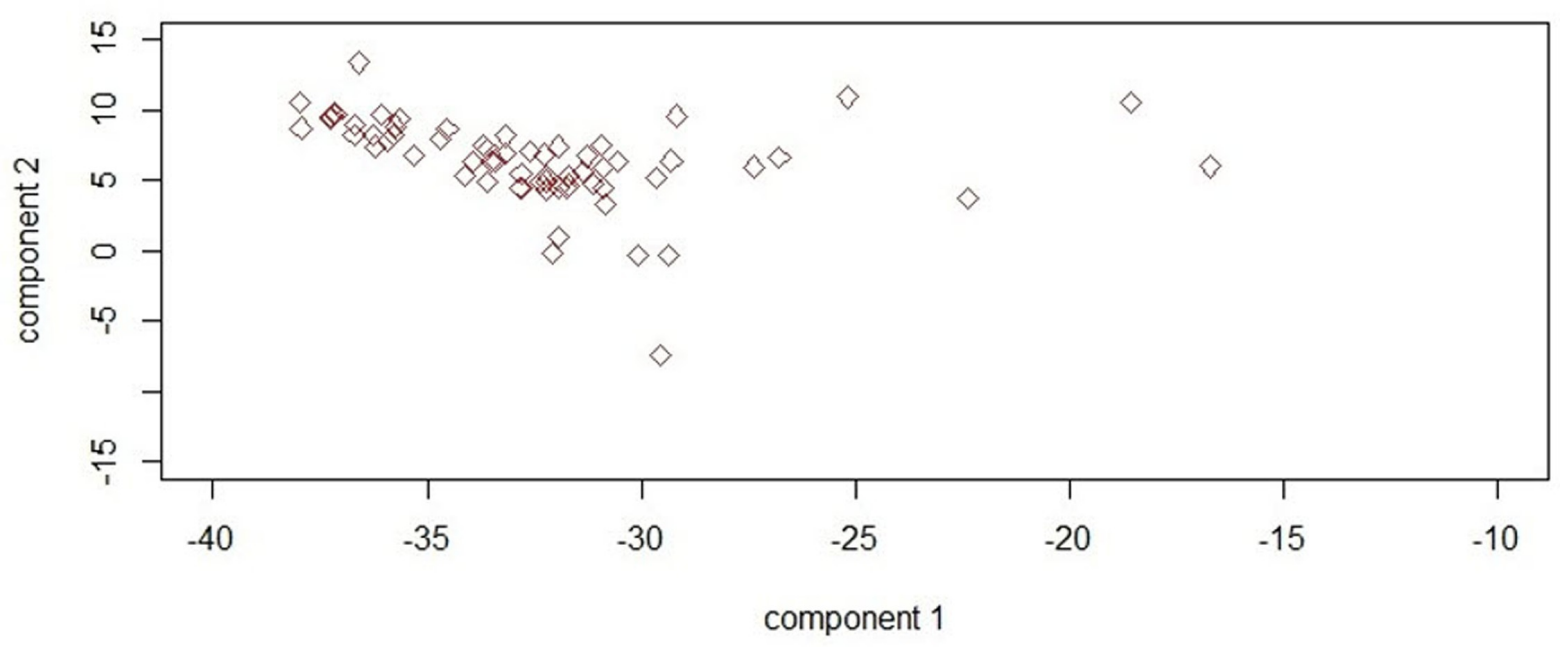
Principal component analysis plot showing the relationship between the first two principal components in the studies population.

Quality control was performed by Plink software version 9.1 beta [/www.cog-genomics.org/plink9.1], and the SNPs were refined as follows: Animals and SNPs call rates with a read rate of less than 95% and minimum allelic frequency of less than 0.01 were excluded. Also, SNPs that were not in Hardy-Weinberg equilibrium (P-value < 1e^-6^) or whose genetic position was not known were excluded from the final analysis [9]. A principal component analysis (PCA) plot of samples was drawn to study the genetic structure of the studied population using the R program. To this end, the PCA was implemented using a genomic relationship matrix constructed with all SNPs, and the first two principal components were used to visualize the population structure (Fig 3).

In the present study, GC correction and very cautious false discovery rate (FDR) correction were also investigated to minimize false results. GC and FDR are statistical methods used in multiple hypothesis testing to correct for multiple testing and have more ability to find accurate results [23].

SNPs were arranged according to chromosome number and coordinate, and their output was saved in tab-delimited and txt format. The Manhattan plots were drawn for each trait by Haploview, and then, for better resolution, their Manhattan and Q-Q plots were drawn by the R program. The Manhattan plot is used appropriately to show the SNPs based on P, GC, and FDR (-log(P)) values that are corrected by p-values. In addition, in the case of SNPs with high corrected p-values (FDR) and corrected p-values (GC) of more than five, they are reported as suggested markers for the desired trait (significant markers) because with conservative tests such as Bonferroni or FDR, a significant effect remains on traits. Also, the GC values of three to five are considered as suggested so that they can be used in future studies.

Different databases were used to identify whether there are candidate genes associated with the studied traits in the genomic regions identified by significant SNPs. The BLAST analysis was applied using different databases such as BioMart-Ensembl [www.ensembl.org/biomart], Ensembl [www.ensembl.org], and NCBI [www.ncbi.nlm.nih.gov/] to identify candidate genes mapped to the genome. The Ensembl database was also used to investigate the biological pathways involved for the identified candidate genes to examine the ontology of genes (including biological processes, molecular functions, and cellular components) [www.ensembl.org]. Finally, identified SNPs were aligned and compared with the reference sequences in the NCBI database by the BLAST method [www.ncbi.nlm.nih.gov/].

## Results

The plink outputs for all traits are given as follows: The number of markers, the number of individuals with non-missing phenotypes, and the number of males and females were 42001, 68 heads, 26 heads, and 42 heads, respectively. The total genotyping rate in the remaining individuals was 0.996576 and after frequency and genotyping pruning, number of SNPs was 41917. Genomic inflation factors for abdomen depth, head width, hip width, and withers height were 1.0778, 1.1239, 1, and 1.1419, respectively. The results of PCA indicated that the first two principal components explained 23.55 and 10.59% of the total genotypic variation, respectively.

### Abdomen depth

**Fig 4:**
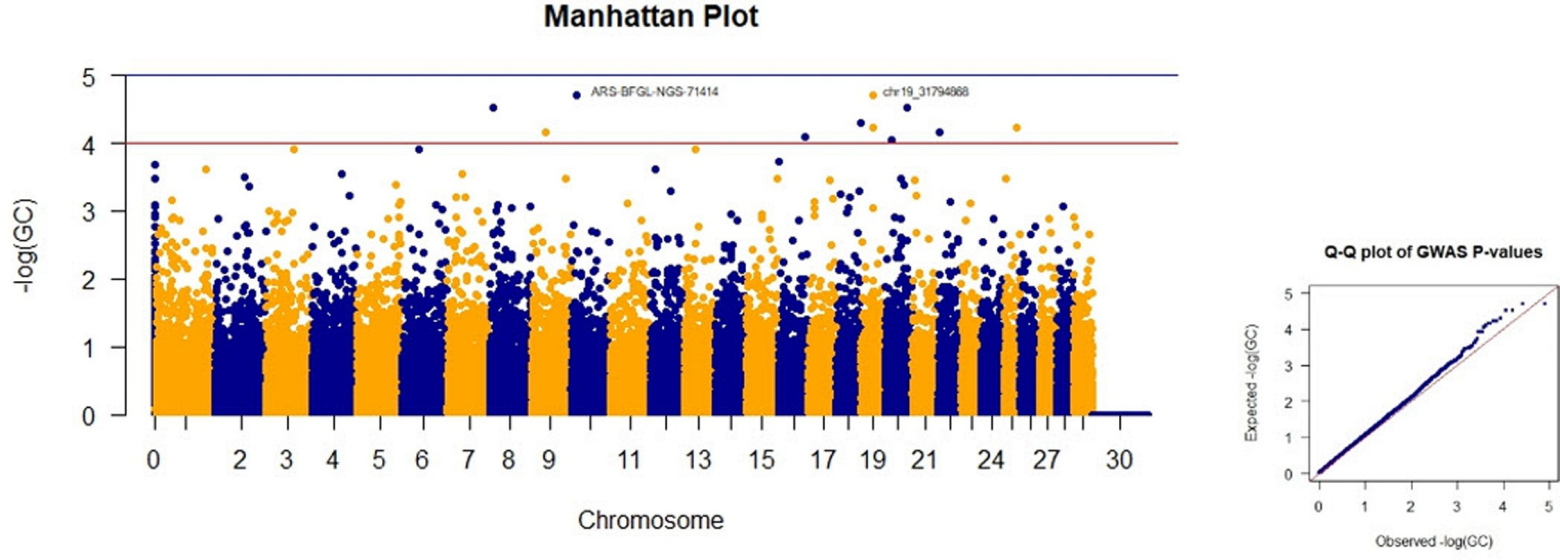
Manhattan and Q-Q plots of GWAS based on the negative logarithm of GC-corrected p-values for abdomen depth.

Four SNPs on chromosomes 19, 10, 8, and 20 had the highest p-values for abdominal depth (Table 2). Although the three SNPs on chromosomes 8, 10, and 19 are close to the significance level, they have a p-value of less than five, and they were not considered significant markers. The Manhattan plots of GWAS based on the the negative logarithm of GC-corrected p-values, negative logarithm of uncorrected p-values, and the negative logarithm of FDR-corrected p-values for abdomen depth are given in Figs 4 and S1 and S2, respectively.

**Table 2:** Single nucleotide polymorphisms associated with abdominal depth which had the lowest p-values.

SNP	CHR+POS.	p-value	GC	FDR
ARS-BFGL-NGS-71414	10.13162750	0.00001	0.00002	0.1581
chr19_31794868	19.31794868	0.00001	0.00002	0.1581
ARS-BFGL-NGS-26667	8.8912792	0.00001	0.00003	0.1581
ARS-BFGL-NGS-81723	20.59463186	0.00002	0.00003	0.1581

### Head width

**Fig 5:**
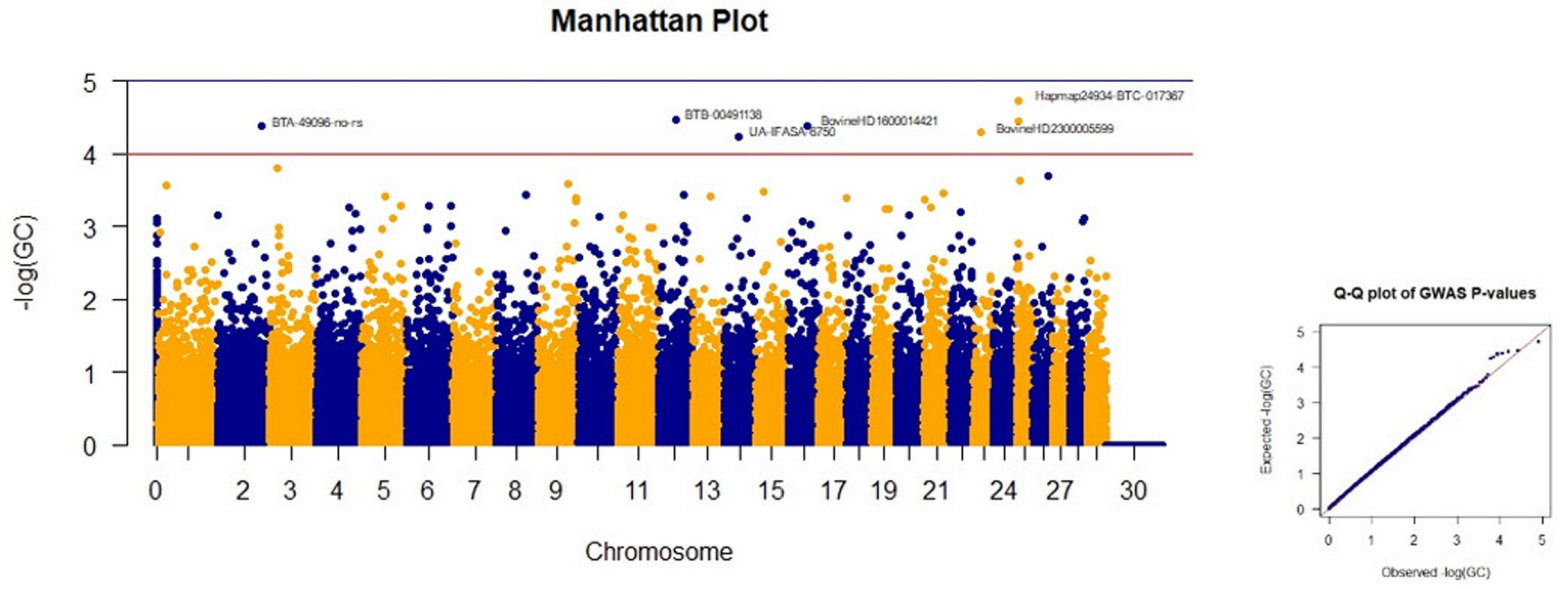
Manhattan and Q-Q plots of GWAS based on the negative logarithm of GC-corrected p-values.

One SNP on chromosome number 25 had the highest p-values for head width (Table 3)**.** As indicated in Table 3, comparing the Manhattan plots based on P, GC, and FDR values showed that there were SNPs with high values. Since p-values are less than 5, they were not considered significant markers**.** The Manhattan plots of GWAS based on the the negative logarithm of GC-corrected p-values, negative logarithm of uncorrected p-values, and the negative logarithm of FDR-corrected p-values for head width are given in Figs 5 and S3 and S4, respectively.

**Table 3:** Single nucleotide polymorphisms associated with head width which had the lowest p-values.

SNP	CHR+POS	p-value	GC	FDR
Hapmap24934-BTC-017367	25.3148958	0.00002	0.00002	0.3461
BTB-00491138	12.45731646	0.00003	0.00003	0.3461
Hapmap27498-BTC-017275	25.3125095	0.00004	0.00004	0.3461
BovineHD1600014421	16.51883201	0.00004	0.00004	0.3461
BTA-49096-no-rs	2.116506798	0.00004	0.00004	0.3461
BovineHD2300005599	23.21325696	0.00005	0.00005	0.3461
UA-IFASA-6750	14.40142442	0.00006	0.00006	0.3461

### Hip width

**Fig 6:**
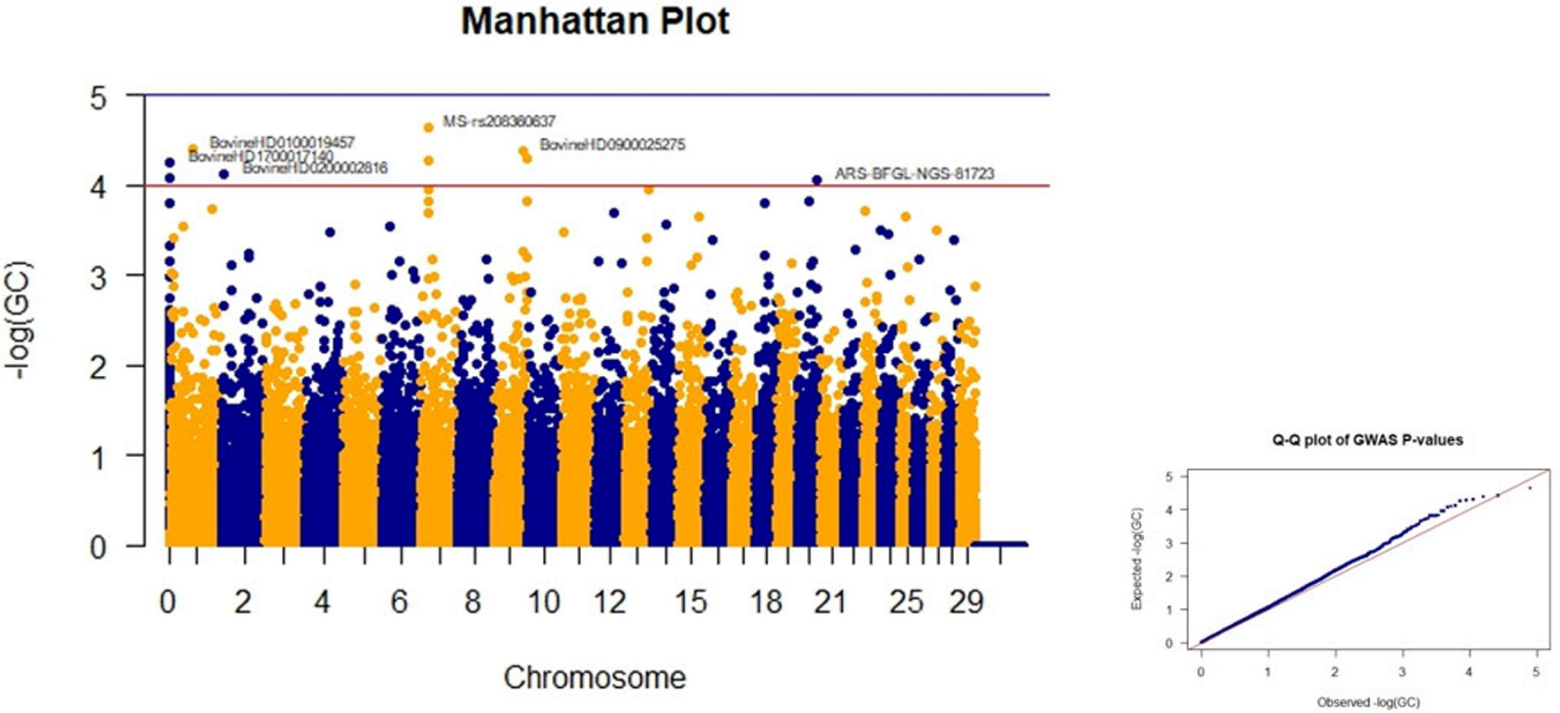
Manhattan and Q-Q plots of GWAS based on the negative logarithm of GC-corrected p-values for hip width.

One SNP on chromosome 7 had the highest p-value for hip width. Comparing the Manhattan plots with the values of P, GC, and FDR, polymorphisms with high values were also observed (Table 4). The first SNP with a p-value greater than five is considered a significant marker (Table 4). The Manhattan plots of GWAS based on the the negative logarithm of GC-corrected p-values, negative logarithm of uncorrected p-values, and the negative logarithm of FDR-corrected p-values for hip width are given in Figs 6 and S5 and S6, respectively.

**Table 4:** Single nucleotide polymorphisms associated with hip width which had the lowest p-values.

SNP	CHR+POS	P	GC	FDR
MS-rs208360637	7.23882024	0.00001	0.00002	0.09463
BovineHD0100019457	1.68834492	0.00001	0.00004	0.09463
BovineHD0900025275	9.89548854	0.00001	0.00004	0.09463
BovineHD0900030355	9.103599311	0.00001	0.00005	0.09463
BovineHD0700006521	7.23786666	0.00001	0.00005	0.09463

### Withers height

**Fig 7:**
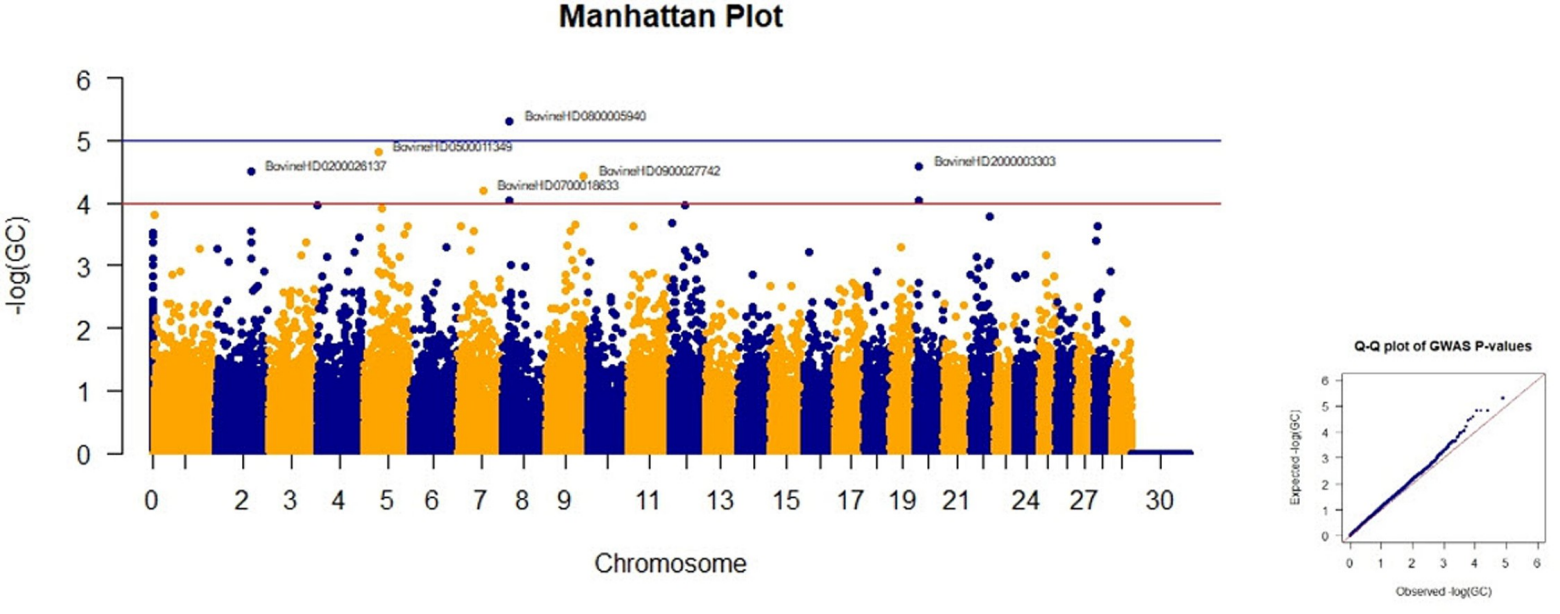
Manhattan and Q-Q plots of GWAS based on the negative logarithm of GC-corrected p-values for withers height.

Two SNPs on chromosomes 5 and 8 had the highest p-values ​​for withers height. Comparing the Manhattan plots based on P, GC, and FDR values showed that there were some other SNPs which are considered indicators implying that the first two SNPs had significant values (Table 5). The Manhattan plots of GWAS based on the the negative logarithm of GC-corrected p-values, negative logarithm of uncorrected p-values, and the negative logarithm of FDR-corrected p-values for withers height are given in Figs 7 and S7 and S8, respectively.

**Table 5:** Single nucleotide polymorphisms associated with withers height which had the lowest p-values.

SNP	CHR+POS	P	GC	FDR
BovineHD0800005940	8.19068939	0.00000	0.00000	0.05959
BovineHD0500011353	5.39624311	0.00001	0.00002	0.05959
BovineHD0500011352	5.39615494	0.00001	0.00002	0.05959
BovineHD0500011349	5.39605647	0.00001	0.00002	0.05959
BovineHD2000003303	20.10462408	0.00001	0.00003	0.083
BovineHD0200026137	2.92028000	0.00001	0.00003	0.08551
BovineHD0900027742	9.97265015	0.00001	0.00004	0.08862

## Discussion

While the population growth rate and economic pressure impose changes in conventional agricultural systems, biodiversity has been decreasing quickly. As one of the chief items of biodiversity, animal genetic resources fulfill the growing demand for food and agriculture. A native animal has always been a component of a specific environment. Such animals play a role in the chain of events for feeding or habitat and have developed with the environment and accommodated to it. Native animals generate a complex web of life. Their loss can quickly influence the entire ecosystem. Native animals are valuable for their roles in healthy ecosystems, supplying economic benefits, and their good adaptation to harsh local environmental situations. Therefore, there is an urgent necessity to conserve the genetic resources of native animals, many of which are at extinction risk. The current study is the first report on GWAS for some biometric traits in NCG, as a cattle breed that is under extinction risk. The main limitation of this study would be the low number of samples used for GWAS. Still, the authors did their best to sample from all accessible pure NCG, which had a valid pedigree with the least genetic relationship among them. Therefore, it was not possible to get a greater sample size in this study.

NCG is one of the most important sources of meat and milk in Guilan province and is also used as working and draft animals in some parts of the province. These cattle have characteristics such as resistance to harsh weather conditions, inadequate nutrition, and various diseases. NCG has high genetic diversity that could be identified using SNP analysis. This high genetic diversity is possibly due to their history of breeding and use in different areas of their habitat. Furthermore, due to the high level of genetic diversity present in NCG, they can be used as a valuable resource in genetic programs. By using SNP analysis and identifying animals with desirable traits, cattle with specific characteristics can be selected, and they can be used as a valuable resource in genetic programs to improve production traits.

### Abdomen depth

Abdomen depth can be an indicator of the volume of abdomen muscles and is considered one of the important traits in meat production in cattle. Given the characteristics of NCG, such as resistance to harsh weather conditions, inadequate nutrition, and various diseases, it can be expected that these cattle have an good abdomen depth for meat production. By using SNP analysis and identifying samples with desirable traits, cattle with high and proper abdomen depth for meat production can be selected, and improvements can be made in the meat yield of these cattle. Therefore, abdomen depth can be considered one of the important traits for meat production in NCG, and by using SNP analysis, improvements can be made in the meat production of this breed.

The ARS-BFGL-NGS-71414 SNP is located at a distance of 1000 bp from the multiple epidermal growth factor-like domains 11 (*MEGF11*) gene at position 10:12763643–13171387, which encodes the Multiple EGF-like domains 11 protein. Chiu et al. [24] demonstrated that upregulation of *MEGF11* gene expression is involved in the mechanism by which the recurrence of Triple Negative Breast Cancer (TNBC) occurs. The aim was to elucidate the role of *MEGF11* expression in TNBC cells, both *in vitro* and *in vivo*, and human tissue. Following *MEGF11* gene knockdown (ΔMEGF11) or over-expression in MDA-MB-231 and MB-468 cells, cell growth and chemokine gene expression were evaluated. In vivo, tumor growth of implanted human TNBC cells and the number of circulating 4T1 mouse tumor cells were measured. There was a significant decrease in cell growth via inhibition of AKT, NF-kB, CREB, and AP-1 activation in ΔMEGF11 MDA-MB-231 and 468 cells. This also resulted, *in vivo*, in the suppression of tumor growth and a decrease in the number of mice circulating 4T1 breast cancer cells. Surprisingly, overexpression of *MEGF11* upregulated the expression of various chemokines and proinflammatory cytokines via AKT activation, but there was no increase in cell proliferation. *MEGF11* was found to cross-talk positively with IL-17A signaling. Patients with tumors that over-expressed *MEGF11* had a poorer prognosis. They conclude that MEGF11 plays an important role in tumor survival and that overexpression of MEGF11 induces both a cytokine and a chemokine cascade, which will favor the tumor microenvironment in terms of distant metastasis. *MEGF11* might be a potential therapeutic target for preventing TNBC recurrence [24].

The *MEGF11* gene regulates diurnal gain and immune response to mastitis in cattle [25, 26]. Another study observed different expressions of *MEGF11* in the longissimus dorsi muscle of two different breeds of cattle in response to low-energy diets [26]. This finding is consistent with GWAS results in pigs suggesting *MEGF11* as a potential candidate gene for feed efficiency [27, 28]. Furthermore, *MEGF11* was significantly associated with height in buffalo [28]. The biological pathway of *MEGF11* is given in S9 Fig. The expression of this gene in different organs in the Atlas is shown in S10 Fig.

At a distance of 1000 from the chr19_31794868 SNP, that is, at 19:31736796–31824993, there is the Heparan sulfate glucosamine 3-O-sulfotransferase 3A1 (*HS3ST3A1*) gene, which encodes Sulfotransferase protein. The biological pathway of the *HS3ST3A1* gene is shown in S11 Fig. *HS3ST3A1* gene expression can be seen in the kidney, liver, and lung organs in the Atlas (S12 Fig).

The ARS-BFGL-NGS-26667 SNP is located in the Methionine Sulfoxide Reductase A (*MSRA*) gene region 8:8746888–9177947, which encodes Peptide-methionine (S)-S-oxide reductase protein.

Heparan sulfate biosynthetic enzymes are key components in producing numerous delicate structures of heparan sulfate that perform numerous biological activities. The enzyme encoded by this gene is a member of the family of heparan sulfate biosynthetic enzymes. It is a type II integral membrane protein with heparan sulfate glucosaminyl 3-O-sulfotransferase activity. The sulfotransferase domain of this enzyme is very similar to the heparan sulfate D-glucosaminyl 3-O sulfotransferase domain, and these two enzymes sulfate the same disaccharide. This gene is widely expressed and has the highest expression in the liver and placenta [29].

MsrA expression was downregulated in MsrA-/- mice. MsrA silencing was shown to produce severely injured motor coordination, increased expressions of Iba1, TNF-α, IL-1β, ROS, and NOX2, and extent of ERK, p38, IκBα, and p65 phosphorylation, but reduced SOD activity. The study suggests that the Tat-MsrA fusion protein can prevent the cellular inflammatory response and subsequent demyelination through negative regulation of the NOX2-MAPKs/NF-κB signaling pathway [13].

The relationship between the *MSRA* gene and the Heat Shock Protein Family E (Hsp10) Member 1 (*HSPE1*) gene can be seen in S13 Fig [https://genemania.org/]. The *HSPE1* gene plays an essential role in the destruction, development, and mobility of muscle cells [30].

The biological pathway of the MSRA gene is given in S14 Fig. *MSRA* gene expression has been reported in the Atlas medium skeletal muscle (S15 Fig). Also, this gene has moderate expression in many organs, such as the brain, intestine, liver, lung, spleen, and testis.

### Head width

The head width in cattle refers to the meat width of their head. In NCG, the width of the head may not directly affect meat production, but it can be used as one of the breed indices and also quality measurement indices for meat. Since the width of the head is related to the breed of cattle as well as their meat weight, cattle with a broader head may have a higher meat weight. Also, generally, cattle with a more robust body structure and better body indices have the highest meat production efficiency. Therefore, in the evaluation of NCG, head width can be used as one of the indices to measure their breed value and meat quality. However, to ensure the accuracy and validity of these evaluations, more precise physiological and genetic tests and analyses are required.

Cyclic adenosine monophosphate Response Element Binding protein Binding Protein (*CREBBP*) gene is located in the region of 20:2757905–3156816, at a distance of 1000 bp from Hapmap24934-BTC-017367 SNP, which encodes the Histone acetyltransferase protein. This gene is involved in cell response to UV, chromatin organization, histone acetylation, histone glutamine methylation, negative regulation of transcription by RNA polymerase II, N-terminal peptidyl lysine acetylation, positive regulation of transcription by RNA polymerase II, positive regulation of the growth factor beta receptor signaling pathway, B Protein stability, response to chemicals, the rhythmic process is involved. Its biological pathway is as follows in S16 Fig.

Hapmap27498-BTC-017275 SNP of the tumor necrosis factor receptor associated protein 1 (*TRAP1*) gene is located in the region of 25:2998564–3049693, which encodes a heat shock protein 75 kDa, mitochondrial. This gene is involved in the negative regulation of cellular respiration, protein folding, and the weakening of translation. In addition, heat shock protein 75 (*HSP75*), also known as TRAP1, has been reported as a candidate gene for regulating body temperature in cattle [31].

S17 Fig shows the relationship between the *TRAP1* gene and other genes, especially the activator of HSP90 ATPase activity 1 (*AHSA1*) gene, which was identified in studies with several Phospholipase A2 Group IIA (*PLA2G2A*), parkin RBR E3 ubiquitin protein ligase (*PARK2*), Zinc Finger Protein 410 (*ZNF410*), Pyruvate kinase isozymes M2 (*PKM2*), Mitogen-Activated Protein Kinase Kinase 3 (*MAP2K3*), Phospholipase C Delta 3 (*PLCD3*), Phospholipase C delta 1 (*PLCD1*), Rho Associated Coiled-Coil Containing Protein Kinase 1 (*ROCK1*), and Activator of HSP90 ATPase Activity 1 (*AHSA1*) genes affect meat fat content in Ankole cattle [32]. The biological path of this gene is shown in S18 Fig. Moderate gene expressions have been reported in skeletal muscle, spleen, testis, liver, heart, colon, and brain (S19 Fig).

### Hip width

The hip width of NCG can be used as one of the breed indices and also could be considered a quality measurement criterion for meat production. The correlation between the width of the hip and meat production may be due to the relationships between the width of the hip and the weight of meat and the body composition of the cattle. Cattle with a broaser hip may have a higher weight and generally have the highest meat production efficiency with a more muscular body composition. Therefore, in the evaluation of NCG, the width of the hip can be used as one of the indices to measure their breed value and meat quality.

At a distance of 100,000 from the MS-rs208360637 SNP, the Chondroitin Sulfate Synthase 3 (*CHSY3*) gene is located in the region of 7:23970491–24269751, which encodes Hexosyltransferase protein. This gene is involved in the biosynthetic process of chondroitin sulfate. In addition, it is one of the candidate genes associated with important economic characteristics that have been identified, such as three genes (calmodulin 2 (*CLSTN2*), Dihydropyrimidine dehydrogenase (*DPYD*), *CHSY3*) and are related to the quality of meat and fatty acids in LFC cattle (native Chinese cattle). *CLSTN2* has an essential role in promoting adipocyte proliferation in visceral adipose tissue and subcutaneous fat and is associated with mammalian obesity [33]. *DPYD* can increase marbling fat [34]. *CHSY3* plays an important role in meat tenderness [35]. Overall, these genes may explain the superior meat quality of local cattle in southern China. Its biological pathway is presented in S20 Fig.

At a distance of 1000 from the SNP named BovineHD0100019457, the Kalirin RhoGEF Kinase (*KALRN*) gene is located at a distance of 1:68641297–68948570, which encodes the Kalirin RhoGEF kinase protein. This gene is involved in axon guidance and central nervous system development. Its biological pathway (S21 Fig).

### Withers height

The presence of withers in NGC is a significant trait that can be used as an indicator of their meat production potential. This trait is rare in most native male cattle and is also rarely seen in females. The meat of withers has 40 to 60 percent fat and is very tasty, as it is often sold at almost double the price. The ability to produce high-quality and palatable meat in NGC can have a significant impact on meat prices and therefore improve livestock profitability. In general, traits such as the presence of bone in the sirloin region can be used as an indicator to increase meat production potential in cattle. Since the production of quality and marketable meat can improve livestock profitability, the use of cattle with positive traits can be considered an effective solution to increase productivity and income in the livestock industry.

At a distance of 10000 bp from the SNP named BovineHD2000003303, the Cyclin Dependent Kinase 7 (*CDK7*) gene is located at the distance of 20:10431993–10461278, which encodes the protein Cell division protein kinase 7. This gene is involved in the phosphorylation of the C-terminal domain of RNA polymerase II, positive regulation of transcription by RNA polymerase II, protein phosphorylation, protein stabilization, regulation of the G1/S transition of the mitotic cell cycle, and initiation of transcription from the RNA polymerase II promoter.

In the research of Pan et al. [36], 185 *CDK* genes were identified and grouped into eight distinct groups in the cattle family, which show extensive homology. General gene expression analysis in different bovine tissues and specific expression analysis during adipocyte differentiation showed that *CDK4*, *CDK7*, *CDK8*, *CDK9*, and *CDK14* might be involved in bovine adipocyte differentiation. The results provide a basis for further study to determine the role of the *CDK* gene family in regulating the differentiation of fat cells, which helps improve beef quality [36]. Its biological path is indicated in S22 Fig. This gene is expressed in the spermatid and 101 other tissues. At a distance of 35000 from the SNP named BovineHD0900027742, the PRKN gene is located in the region 9:96944026–98134921, which encodes the protein Parkin RBR E3 ubiquitin-protein ligase. This gene is involved in the motor behavior of adults, mitochondrial autophagy, cellular response to toxic substances, dopamine metabolic process, dopamine uptake in synaptic transmission, ubiquitin chain polymerization, mitochondrial to lysosome transfer, mitophagy, negative regulation of genome replication by the viral host, Negative regulation of actin filament bundle assembly, negative regulation of neuron intrinsic apoptosis signalling pathway caused by endoplasmic reticulum stress, negative regulation of exosomal secretion, negative regulation of gene expression, negative regulation of glucokinase activity, negative regulation of insulin secretion, negative regulation of intraluminal vesicle formation, negative regulation Intrinsic apoptotic signalling pathway by mediator of p53 class, negative regulation of neuron intrinsic apoptosis signalling pathway caused by oxidative stress, negative regulation of primary amine oxidase activity, negative regulation of protein phosphorylation, negative regulation of reactive oxygen species metabolic process, negative regulation of cytochrome c release from Mitochondria, negative regulation of spontaneous neurotransmitter secretion, negative regulation of transcription by RNA polymerase II, norepinephrine metabolic process, parkin-mediated stimulation of mitophagy in response to mitochondrial depolarization, positive regulation of Mitochondrial phagy, upregulation of dendrite expansion, upregulation of DNA binding, upregulation of gene expression, upregulation of I-kappaB kinase/NF-kappaB, upregulation of mitochondrial fusion, upregulation of neurotransmitter uptake, upregulation of Ubi-dependent protein catabolic process Proteasomal chitin, upregulation of protein binding, upregulation of linear protein polyubiquitination, upregulation of protein replacement to the membrane, upregulation of transcription by RNA polymerase II, upregulation of tumor necrosis factor-mediated signalling pathway, dependent protein catabolic process Proteosome-mediated ubiquitination, protein autoubiquitination, protein instability, K11 protein-linked ubiquitination, K48 protein-linked ubiquitination, K63 protein-linked ubiquitination, Ubi K6 protein-related ubiquitination, protein replacement in mitochondria, protein monoubiquitination, protein polyubiquitination, protein stabilization, regulation of apoptosis process, regulation of cellular response to oxidative stress, regulation of dopamine metabolic process, regulation of mitochondrial membrane potential, Mitochondrial organization regulation, response to S Endoplasmic reticulum fear, startle response, synaptic transmission, glutamatergic, ubiquitin-dependent protein catabolic processes are involved.

Also, *PRKN*, also known as *PARK2*, functions are related to fat metabolism and adipogenesis. Significantly, it is a strong candidate for lipid regulation in chicken [36]. Its biological path is presented in S23 Fig. This gene is expressed in the gluteal muscle and 96 other tissues. Its expression level is moderate in skeletal muscle, testicle, spleen, kidney, liver, heart, kidney, colon, and brain (S24 Fig).

## Conclusions

This is the first report of GWAS on some biometric traits in NCG. This study identified significant SNPs and candidate genes associated with meat production traits such as abdominal depth, head width, hip width, and withers height in NCG. These genes are involved in muscle growth, meat tenderness, differentiation of fat cells, fat metabolism, and adipogenesis, and can contribute to the production of meat quantity and quality. The identification of these genes provides valuable insights into the genetics of NCG and can be used for the improvement of meat production traits in this native cattle breed. Because this breed is currently under extinction risk, the results of this study could be implemented in the genetic programs for the preservation of this native breed of cattle.

## Supporting information

S1 Fig Manhattan plot of GWAS based on the negative logarithm of uncorrected p-values for abdomen depth. (JPG)

S2 Fig Manhattan plot of GWAS based on the negative logarithm of FDR-corrected p-values for abdomen depth. (JPG)

S3 Fig Manhattan plot of GWAS based on the negative logarithm of uncorrected p-values for head width. (JPG)

S4 Fig Manhattan plot of GWAS based on the negative logarithm of FDR-corrected p-values for head width. (JPG)

S5 Fig Manhattan plot of GWAS based on the negative logarithm of uncorrected p-values for hip width. (JPG)

S6 Fig Manhattan plot of GWAS based on the negative logarithm of FDR-corrected p-values for hip width. (JPG)

S7 Fig Manhattan plot of GWAS based on the negative logarithm of uncorrected p-values for withers height. (JPG)

S8 Fig Manhattan plot of GWAS based on the negative logarithm of FDR-corrected p-values for withers height. (JPG)

S9 Fig Identification of MEGF11 gene in the range of 10:12763643–13171387 adjacent to ARS-BFGL-NGS-71414 SNP. (JPG)

S10 Fig MEGF11 gene expression in different parts of the cattle’s body. (JPG)

S11 Fig Identification of the HS3ST3A1 gene in the range of 19:31736796–31824993, adjacent to the chr19_31794868 SNP. (JPG)

S12 Fig HS3ST3A1 gene expression in different parts of the cattle’s body. (JPG)

S13 Fig MSRA gene relation with other genes. (JPG)

S14 Fig Identification of MSRA gene in the range of 8:8746888–9177947 adjacent to ARS-BFGL-NGS-26667 SNP. (JPG)

S15 Fig MSRA gene expression in different parts of the cattle’s body. (JPG)

S16 Fig Identification of CREBBP gene in the range of 20:2757905–3156816 adjacent to Hapmap24934-BTC-017367 SNP. (JPG)

S17 Fig The relationship between the TRAP1 gene and other genes. (JPG)

S18 Fig Identification of TRAP1 gene in the range of 25:2998564–3049693 adjacent to Hapmap27498-BTC-017275 SNP. (JPG)

S19 Fig TRAP1 gene expression in different parts of the cattle’s body. (JPG)

S20 Fig Identification of CHSY3 gene in the range of 7:23970491–24269751 adjacent to MS-rs208360637 SNP. (JPG)

S21 Fig Identification of KALRN gene in the range of 1:68641297–68948570 adjacent to Bovine SNP HD0100019457. (JPG)

S22 Fig Identification of CDK7 gene in the range of 20:10431993–10461278 adjacent to BovineHD2000003303 SNP. (JPG)

S23 Fig PRKN gene expression in different parts of the cattle body Identification of PRKN gene in the range of 9:96944026–98134921 adjacent to Bovine SNP HD0900027742. (JPG)

S24 Fig PRKN gene expression in different parts of the cattle body. (JPG)
